# Clonal replacement by a P1-1/ST3 lineage in pediatric *Mycoplasma pneumoniae*, Jinan, China, 2021–2024

**DOI:** 10.3389/fcimb.2025.1732239

**Published:** 2026-01-09

**Authors:** Ming Fang, Xiao Wang, Xiaolin Yu, Jianmei Yu, Lu Yuan, Shuang Wang, Haijian Zhou, Ti Liu, Huaning Zhang, Zengqiang Kou

**Affiliations:** 1 Shandong Center for Disease Control and Prevention, Shandong Province Key Laboratory of Intelligent Monitoring, Early Warning, and Prevention of Infectious Diseases, Jinan, China; 2 Department of Pediatrics, The Affiliated Hospital of Shandong University of Traditional Chinese Medicine, Jinan, Shandong, China; 3 Infection Management Department, Neijiang Second People's Hospital, Neijiang, Sichuan, China; 4 National Institute for Communicable Disease Control and Prevention, Chinese Center for Disease Control and Prevention, National Key Laboratory of Intelligent Tracking and Forecasting for Infectious Diseases, Beijing, China

**Keywords:** clonal replacement, genomic epidemiology, macrolide resistance, *Mycoplasma pneumoniae*, pediatric pneumonia, whole-genome sequencing

## Abstract

**Introduction:**

After a prolonged lull during COVID-19 non-pharmaceutical interventions, *Mycoplasma pneumoniae* activity re-emerged in 2023 in multiple regions; in China this occurred against a backdrop of very high macrolide resistance. We conducted a retrospective single-center study of pediatric *M. pneumoniae* pneumonia in Jinan, comparing a pre-resurgence period (2021) with 2023–2024.

**Methods:**

Clinical data were linked to whole-genome sequencing of 227 cultured isolates. We assessed lineage composition and relatedness using core-genome phylogenetics and SNP-threshold networks, and compared diversity and pan-genome functional profiles across major clades. Phenotypic antimicrobial susceptibility testing was performed.

**Results:**

The proportion of severe cases increased from 7.4% (2021) to 19.9% (2024). Over the same interval, the P1-1/ST3 lineage rose from 41.9% to 84.0%, displacing previously co-circulating lineages. Core-genome analyses indicated reduced diversity and a compact ST3 cluster within the T1-3R subclade of the P1-type 1 lineage (EC1 clone), alongside a smaller P1-type 2/T2-2 (EC2/ST14) clade. Using a ≤11-SNP threshold, 74% of isolates fell within the largest connected component. Pan-genome comparisons suggested enrichment of replication/recombination/repair functions in T1-3R, whereas canonical adhesion factors and the CARDS toxin were conserved. All isolates carried the 23S rRNA A2063G substitution with phenotypic macrolide resistance, while *in vitro* susceptibility to tetracycline and levofloxacin was retained.

**Discussion:**

The 2023–2024 resurgence coincided with clonal replacement by P1-1/ST3 in a setting of fixed macrolide resistance and an increase in severe pediatric disease. Given the retrospective, culture-based design, this should be interpreted as a temporal association rather than evidence that ST3 intrinsically caused more severe disease. These findings support consideration of non-macrolide agents in similar high-resistance settings and motivate prospective genomic-clinical surveillance.

## 1 Introduction

*Mycoplasma pneumoniae* is a major cause of community-acquired pneumonia (CAP) in children and adolescents, accounting for up to 40% of cases (Brown et al., 2016), particularly among school-aged populations. Its epidemiology is characterized by a distinct cyclical pattern, with regional epidemics typically occurring every 3 to 7 years (Gao et al., 2019). However, the respiratory infectious-disease landscape shifted after the onset of the COVID-19 pandemic in early 2020. Widespread non-pharmaceutical interventions (NPIs)—including mask-wearing, social distancing, and school closures—reduced SARS-CoV-2 transmission and were accompanied by a marked, sustained decrease in other respiratory pathogens, including *M. pneumoniae* (Meyer Sauteur et al., 2022).

This prolonged period of minimal exposure informed the “immunity debt” hypothesis, which posits that reduced natural boosting would increase the number of immunologically naive individuals, especially children (Cohen et al., 2021). With NPI relaxation, this accumulated susceptibility was expected to fuel larger-than-usual resurgences (Edens et al., 2024). This prediction began to materialize in mid-2023, when surveillance systems in Europe and Asia reported a delayed, marked re-emergence of *M. pneumoniae* infections (Meyer Sauteur et al., 2025).

In East Asia—particularly in China—the resurgence intersected with a longstanding challenge: high prevalence of macrolide-resistant *M. pneumoniae* (MRMP). For over a decade, MRMP rates in China have often exceeded 90% (Yan et al., 2024), limiting the empirical effectiveness of first-line agents such as azithromycin and complicating clinical management (Jia et al., 2024). The combination of a more susceptible pediatric population and a highly resistant pathogen created a high-risk setting.

This context raises a key question: was the 2023–2024 *M. pneumoniae* epidemic simply a quantitative rebound into a susceptible population, or did it also reflect a qualitative shift in the pathogen population? In particular, whether increased severity was associated with selection of a lineage with greater transmissibility or virulence remains uncertain. Detailed genomic and clinical characterizations from this period are limited (Hakim et al., 2021). Therefore, this study integrates clinical data with whole-genome sequencing to dissect the resurgence of *M. pneumoniae* in a pediatric cohort in Jinan, China. By comparing the pre-resurgence (2021) and post-resurgence (2023–2024) periods, we aim to elucidate the genomic drivers, population dynamics, and clinical consequences of this pathogen’s return.

## 2 Materials and methods

### 2.1 Study design and patients

We carried out a single-center retrospective cohort study of children ≤16 years with pneumonia attributed to *Mycoplasma pneumoniae*, admitted to the Affiliated Hospital of Shandong University of Traditional Chinese Medicine (Jinan, China) during three epidemic windows: May–December 2021, November–December 2023, and October–December 2024. A total of 798 throat swab specimens were collected (338 in 2021, 174 in 2023, and 286 in 2024). Sampling windows were not identical across years. In 2021, microbiological surveillance was implemented over a broader May–December interval while the program was being established during the early post–COVID-19 control phase, under China’s normalized prevention-and-control and dynamic zero-COVID strategy. In 2023 and 2024, we intentionally focused culture and sequencing on shorter November–December and October–December windows that coincided with the local post-COVID resurgence of *M. pneumoniae* seen in hospital and regional surveillance.

The diagnosis of *M. pneumoniae* pneumonia (MPP) was based on clinical symptoms, lung imaging, and laboratory testing. Pneumonia severity was classified according to the Chinese National Health Commission’s Guidelines for CAP in Children (2019), which define severe pneumonia as the presence of at least one of the following: hypoxemia (oxygen saturation <92% on room air) and/or marked respiratory distress (age-adjusted tachypnea, chest wall indrawing, nasal flaring, grunting, or intermittent apnea); extensive radiographic involvement (consolidation affecting ≥2/3 of a lung, multilobar disease, or complications such as pleural effusion, pneumothorax, atelectasis, necrotizing pneumonia, or lung abscess); or serious extrapulmonary complications including sepsis, septic shock, or acute respiratory failure requiring intensive care support (National Health Commission of the People’s Republic of China, and State Administration of Traditional Chinese Medicine, 2019). The denominator for year-specific proportions (e.g., severe disease rate) was the number of children enrolled with clinically diagnosed MPP in that calendar year, which equals the number of study participants with specimens collected in that year.

### 2.2 Ethical approval

The studies involving human participants were reviewed and approved by the Institutional Review Board of the Shandong Provincial Center for Disease Control and Prevention, Jinan, China (Approval No. 2021-24). The studies were conducted in accordance with local legislation and institutional requirements. Given the retrospective design using de-identified residual clinical specimens and metadata, written informed consent was waived by the IRB in accordance with national research-ethics guidelines.

### 2.3 Microbiological testing

All throat swab specimens were initially screened for *Mycoplasma pneumoniae* using a commercial real-time quantitative PCR (qPCR) kit (Shanghai BioGerm Medical Technology Co., Ltd.), following the manufacturer’s instructions. Specimens that tested positive were subsequently genotyped for P1–1 and P1–2 using a duplex real-time PCR assay as previously described (Zhao et al., 2015). Direct multilocus sequence typing (MLST) was then attempted for all qPCR-positive specimens by PCR amplification and Sanger sequencing of internal fragments of eight housekeeping genes according to the established *M. pneumoniae* MLST scheme (Brown et al., 2015); specimens with insufficient template DNA, typically reflected by high qPCR cycle-threshold (Ct) values, failed amplification and were not assigned sequence types. Concurrently, all PCR-positive specimens were inoculated into Mycoplasma Broth Base (CM1166; Oxoid, Thermo Fisher Scientific) supplemented with Mycoplasma Supplement G (SR0059; Oxoid) for enrichment culture, regardless of clinical severity. Upon indication of growth, aliquots were subcultured onto Mycoplasma Agar Base (CM0401; Oxoid) supplemented with Mycoplasma Supplement G (SR0059) for isolation and monitored for the appearance of typical “fried-egg colonies”.

### 2.4 Whole-genome sequencing and genomic analysis

Genomic DNA was extracted from 2 mL of log-phase Mycoplasma broth culture for each isolate using the QIAamp DNA Mini Kit (Qiagen, Hilden, Germany). Sequencing libraries were prepared using the Nextera XT DNA Library Prep Kit and sequenced on a NextSeq 2000 platform (Illumina, San Diego, CA, USA) to generate 150 bp paired-end reads, achieving a target depth of at least 100× coverage. Raw sequencing reads were processed with Fastp (v1.0.1) to remove adapters and low-quality bases. The resulting high-quality reads were *de novo* assembled into draft genomes using SPAdes (v4.2.0) with default parameters. The quality of the draft assemblies was then assessed using QUAST (v5.0.2) (Jiao et al., 2025). Assemblies were retained for downstream analyses if they had a total length between 0.80 and 0.90 Mb, an N50 of at least 50 kb, no more than 50 contigs longer than 500 bp, and a mean depth of coverage ≥50×; all 227 genomes met these criteria.

The assembled genomes were used for downstream analysis. Multilocus sequence typing (MLST) was conducted using the MLST tool (v2.23.0), with sequence types (STs) assigned according to the scheme hosted on the PubMLST database. To identify macrolide resistance mutations, quality-filtered reads were mapped to the *Mycoplasma pneumoniae* 23S rRNA gene reference sequence (GenBank: X68422.1) using CLC Genomics Workbench (v25.0.3; Qiagen, Aarhus, Denmark). Variants were called from the alignments, and domain V of the 23S rRNA gene was screened for macrolide-resistance–associated substitutions at positions A2063G/C/T, A2064G/C, A2067G, and C2617G/A, as previously described (Wang et al., 2024).

### 2.5 Clade and epidemic-clone assignment

We adopted a six-subclade framework for *Mycoplasma pneumoniae* (T1-1, T1-2, T1-3, T1-3R, T2-1, T2-2) based on recent whole-genome phylogenies and nomenclature proposals (Kenri et al., 2023). Within this scheme, the EC1 and EC2 epidemic clones described in China correspond to the T1-3R and T2–2 subclades, respectively (Li et al., 2024). Reads were mapped to the FH reference genome LR214945.1, and SNPs were called at the EC1/EC2-defining sites reported by Li et al. Isolates matching the EC1 or EC2 SNP profiles were classified as EC1 or EC2. EC status was then combined with P1 type and phylogenetic position to assign P1-type 1 EC1 isolates to the T1-3R (EC1) clade, P1-type 1 non-EC1 isolates to T1-3, and P1-type 2 EC2 isolates to T2-2 (EC2).

### 2.6 Phylogenetic and network analysis

**Figure 1:**
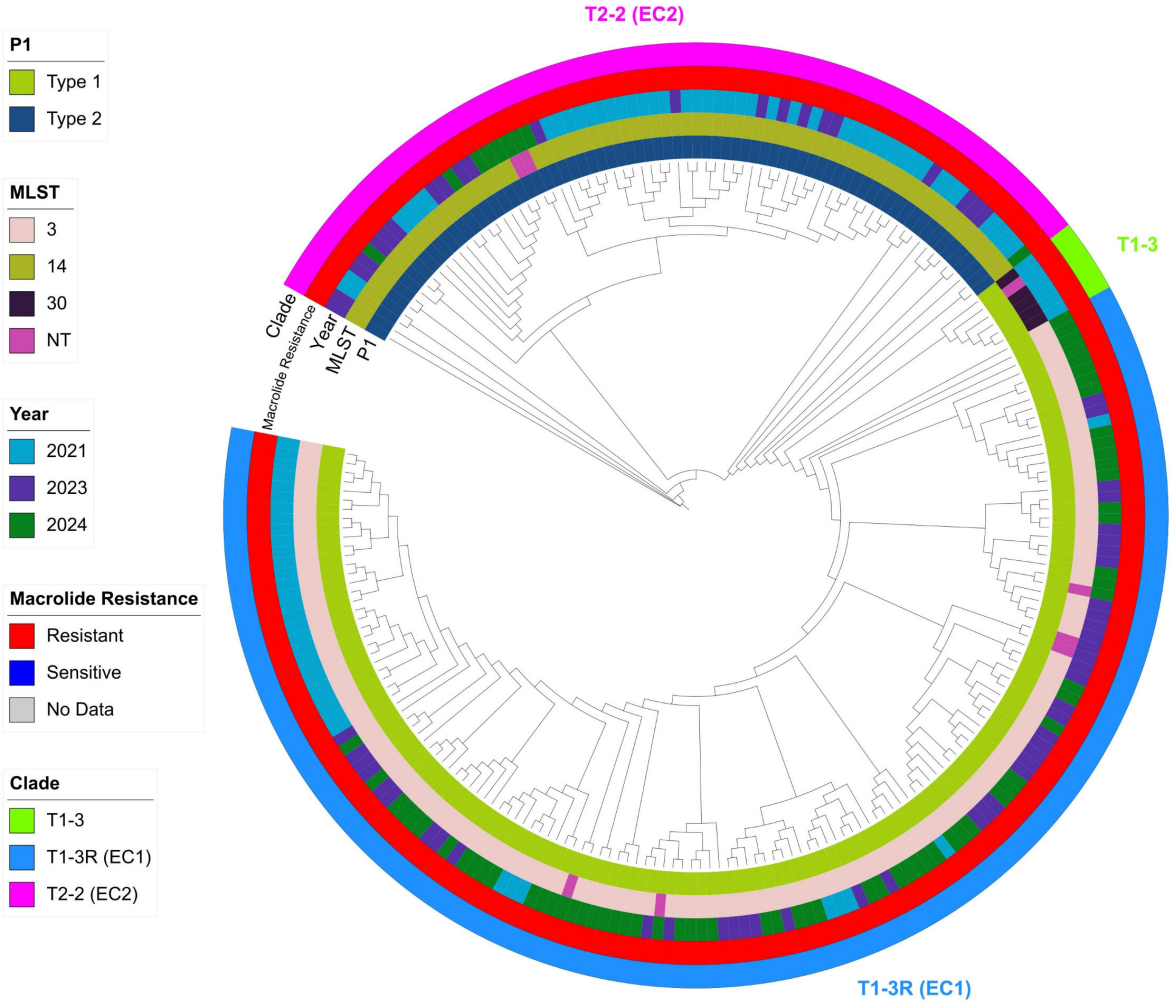
Phylogenetic relationship of 227 *Mycoplasma pneumoniae* isolates from Jinan. Maximum-likelihood tree inferred from recombination-filtered core-genome SNPs using RAxML (GTRGAMMA model, 100 rapid bootstrap replicates), annotated with colored bars for P1 type, MLST sequence type (ST), collection year, macrolide resistance status, and phylogenetic clade. The topology resolves two major clades, the P1-type 1/T1-3R (EC1) clade and the P1-type 2/T2-2 (EC2) clade, and shows evidence of a recent clonal expansion within the T1-3R (EC1) clade during 2023–2024. The monophyly of the T1-3R (EC1) and T1–3 clades, as well as of the overall type 1 lineage (T1-3R + T1-3), is strongly supported (bootstrap 100% for each); to maintain visual clarity, these bootstrap values are summarized here rather than shown on every branch. The tree is displayed as a cladogram (branch lengths not to scale).

**Figure 2:**
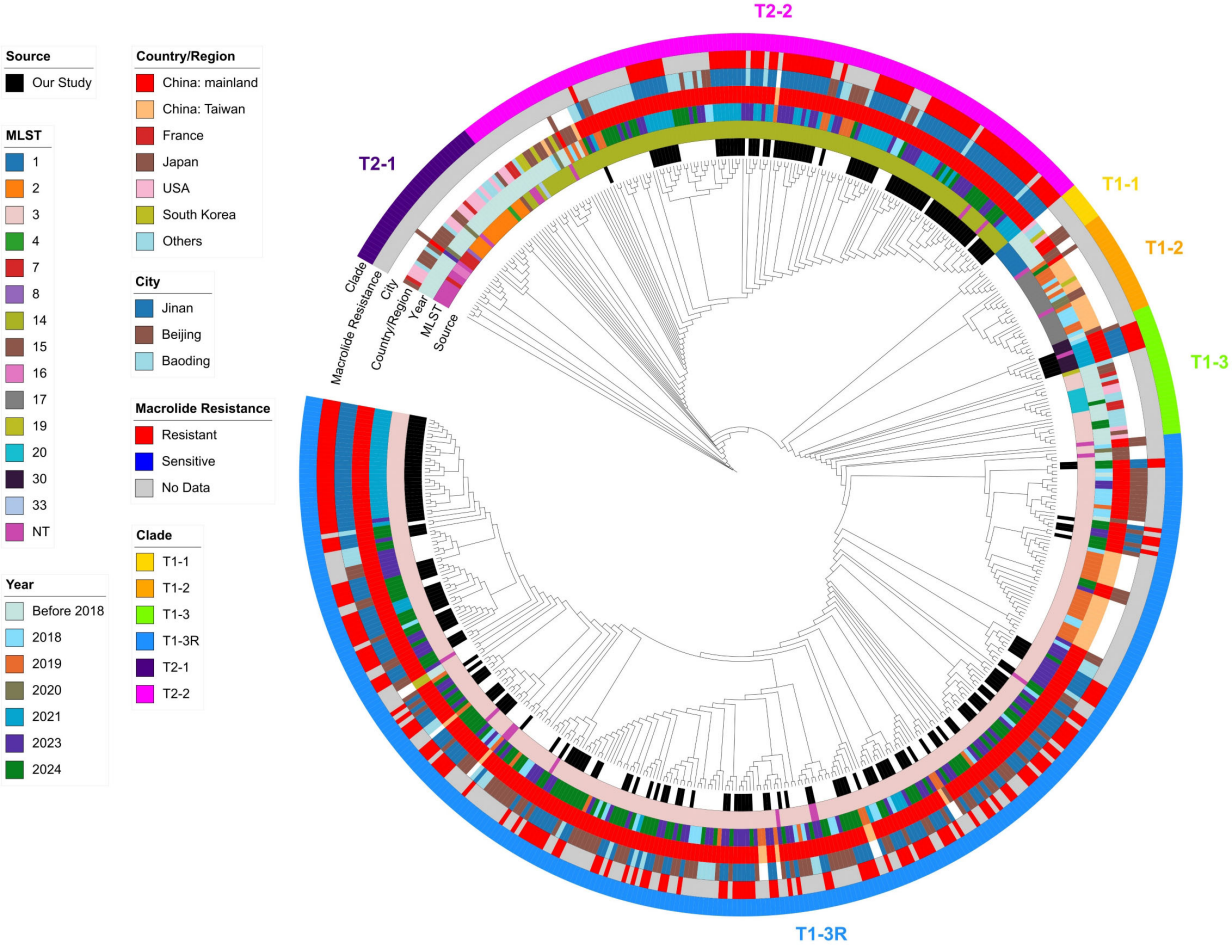
Global phylogenetic context of Jinan *M. pneumoniae* isolates. A circular maximum-likelihood tree showing the phylogenetic relationship between the 227 isolates from this study (Jinan) and 308 global reference strains. Concentric colored rings annotate, from the tree outward, clade assignment, macrolide resistance status, city, country/region, MLST sequence type, sampling year, and data source. The tree shows that Jinan isolates are interspersed within the global diversity of the T1-3R (EC1) and T2-2 (EC2) clades, suggesting connections to regional and global strain populations. The tree is displayed as a cladogram (branch lengths not to scale) for visual clarity.

Reads were mapped to *M. pneumoniae* M129 (NC_000912) using Snippy (v4.6.0) with default quality filters (minimum site depth 10×, minimum mapping quality 60 and minimum base quality 13) to call SNPs and indels and generate the core alignment; core SNPs were extracted with SNP-sites, and recombination was detected and masked with Gubbins (v3.4.3) (Croucher et al., 2015). Pairwise SNP distances were computed with snp-dists, and a maximum-likelihood tree was inferred with RAxML (v8.2.12) under GTRGAMMA using rapid bootstrapping (-f a, 100 replicates). Transmission clusters were explored via a genomic network linking isolates at ≤3 SNPs (putative recent transmission) and, for broader connectivity, ≤11 SNPs, following prior outbreak work (with thresholds evaluated in sensitivity analyses) (Jiao et al., 2025). Trees were visualized in iTOL as cladograms with branch lengths ignored (**Figures 1**, **2**) for improved readability, while all distance-based analyses were performed on the full branch-length trees.

### 2.6 Genome re-annotation, pangenome inference, and differential gene-presence analysis

Draft genomes were re-annotated with Prokka (v1.14.6) using -{{-}}-genus Mycoplasmoides -{{-}}-species pneumoniae -{{-}}-gcode 4 (translation **Table 1** is appropriate for Mycoplasmoides/Mycoplasma) to generate GFF and protein FASTA files. Orthologous clusters and the gene presence/absence matrix were inferred with Panaroo (-i./*.gff -o panaroo_out -{{-}}-clean-mode moderate -a core -{{-}}-aligner mafft -t 4 -{{-}}-remove-invalid-genes), using the default clustering thresholds (minimum amino-acid identity 0.98, protein family identity threshold 0.70 and length difference cutoff 0.98) and a core-genome threshold of 0.95. Cluster-level association between clades (T1-3R (EC1) vs T2-2 (EC2)) and gene presence was tested in R (v4.5.0) using Fisher’s exact test; clusters with nominal *p* < 0.05 were carried forward to Benjamini–Hochberg false discovery rate correction, and those with adjusted *q* < 0.05 were considered significant. Functional annotation of cluster member proteins was performed with eggNOG-mapper (v2.1.13) (DIAMOND mode) against the official eggNOG v5 reference, summarizing COG categories at the cluster level; clusters without any COG assignment were labeled “Unannotated”, whereas clusters assigned to COG category S were labeled “Function unknown”.

**Table 1:** Genotype distribution of *Mycoplasma pneumoniae* isolates, 2021–2024.

Year	Total (n)	P1–1 Subtypes			P1–2 Subtypes	
		ST3	ST30	Untypeable	ST14	Untypeable
2021	86	36 (41.9%)	5 (5.8%)	1 (1.2%)	44 (51.2%)	0 (0.0%)
2023	66	42 (63.6%)	0 (0.0%)	2 (3.0%)	22 (33.3%)	0 (0.0%)
2024	75	63 (84.0%)	0 (0.0%)	3 (4.0%)	7 (9.3%)	2 (2.7%)

Data are presented as n (%), where percentages are calculated relative to the total number of isolates (n) for each year.

### 2.7 Virulence gene identification

Predicted proteins were searched against the Virulence Factor Database (VFDB; http://www.mgc.ac.cn/VFs/) using BLASTp. Matches were accepted only if sequence identity ≥85% and query coverage ≥85%, retaining the top hit per query. Calls were summarized as a binary presence/absence matrix for downstream analyses. For key virulence determinants, we refer to proteins by their conventional names and indicate the corresponding M129 locus tags at first mention.

### 2.8 Antimicrobial susceptibility testing

Antimicrobial susceptibility was determined by broth microdilution and interpreted per CLSI M43-A (macrolides with defined S/R categories; tetracycline and levofloxacin with susceptible-only criteria) (Clinical and Laboratory Standards Institute (CLSI), 2011).

### 2.9 Statistical analysis

Continuous variables were summarized as medians (range) and compared across years (2021, 2023, 2024) using the Kruskal–Wallis test. Categorical variables were presented as counts (%) and analyzed with Pearson’s chi-square or Fisher’s exact test; trends over time were assessed where applicable. Pan-genome associations used Fisher’s exact test with Benjamini–Hochberg FDR control; clusters with nominal p<0.05 and adjusted q<0.05 were considered significant. Two-sided p<0.05 was considered statistically significant. Analyses were performed in R (version 4.5.0).

## 3 Results

### 3.1 Laboratory confirmation and isolate recovery from the clinical cohort

From 2021 to 2024, 798 children with clinically diagnosed MPP were enrolled. The PCR confirmation rate increased from 47.3% (160/338) in 2021 to 75.9% (217/286) in 2024, with an overall rate of 59.8% (477/798) (**Table 2**). Among the 477 PCR-positive specimens, 227 *Mycoplasma pneumoniae* isolates were cultured and subjected to genomic analysis.

**Table 2:** Summary of specimen collection, PCR detection, and bacterial isolation of *Mycoplasma pneumoniae* from 2021 to 2024.

Category	2021	2023	2024	Total
Total specimens collected, n	338	174	286	798
PCR-positive specimens, n (%)	160 (47.3%)	100 (57.5%)	217 (75.9%)	477 (59.8%)
Isolates successfully cultured, n (%)¹	86 (53.8%)	66 (66.0%)	75 (34.6%)	227 (47.6%)

¹The percentage of successful culture was calculated based on the number of PCR-positive specimens.

### 3.2 Escalating disease severity in recent epidemics

Clinical severity of pediatric MPP increased in the post-COVID era despite stable demographics (median age ~7.5 years and similar sex ratios). Across the three annual cohorts, peak temperature and length of stay rose stepwise (38.5→38.8→39.0 °C; 7.0→8.5→9.5 days; **Table 3**). Systemic inflammatory burden increased as well: CRP surged in 2023 and remained above 2021 in 2024 (20.1→47.8→34.7 mg/L), while LDH peaked in 2023 and declined but stayed slightly higher than baseline in 2024 (338→464→349 U/L). Coagulation activity rose (D-dimer 1.05→1.55→1.70 µg/mL), and PCT peaked in 2023 before declining in 2024 yet remained above 2021 levels (1.9→2.9→2.3 ng/mL). Neutrophil fractions were stable, and WBC counts showed no consistent upward trend. Concordantly, the proportion of severe pneumonia increased year-on-year from 7.4% (25/338) in 2021 to 14.9% (26/174) in 2023 and 19.9% (57/286) in 2024 (**Table 3**), indicating a measurable shift toward more severe disease in recent epidemics.

**Table 3:** Demographics, clinical, and laboratory characteristics of pediatric patients with *Mycoplasma pneumoniae* pneumonia by year.

Characteristic	2021 (n=338)	2023 (n=174)	2024 (n=286)
Demographics and Clinical Manifestations			
Male/Female Ratio	1.4/1	1.2/1	1.3/1
Age, years, median (range)	7.5 (1–14)	7.5 (1–14)	7.4 (0.8–14)
Peak fever (°C), median (range)	38.5 (37.3–39.6)	38.8 (37.5–40.0)	39.0 (37.8–40.2)
Length of hospital stay, days, median (range)	7.0 (4–10)	8.5 (5–12)	9.5 (5–14)
Laboratory Findings			
WBC (×10^9^ cells/L), median (range)	11.4 (6.2–16.6)	9.5 (5.4–15.3)	11.2 (5.6–16.8)
Neutrophils (%), median (range)	61.0 (41.7–80.2)	61.3 (43.6–78.9)	61.3 (42.0–80.5)
CRP (mg/L), median (range)	20.1 (1.8–38.3)	47.8 (2.1–93.4)	34.7 (1.4–68.0)
PCT (ng/mL), median (range)	1.9 (0.12–3.62)	2.9 (0.10–5.72)	2.3 (0.18–4.38)
LDH (U/L), median (range)	338 (246–430)	464 (280–648)	349 (220–478)
D-dimer (μg/mL), median (range)	1.05 (0.3–1.8)	1.55 (1.0–2.1)	1.70 (0.2–3.2)
Outcomes			
Severe pneumonia, n (%)	25 (7.4%)	26 (14.9%)	57 (19.9%)

Data are presented as median (range) or n (%).Denominator for yearly percentages: children enrolled with clinically diagnosed MPP in the corresponding year.

CRP, C-reactive protein; LDH, lactate dehydrogenase; PCT, procalcitonin; WBC, white blood cell.

### 3.3 A Rapid genotypic shift toward the P1-1/ST3 clone

At the specimen level, direct P1/MLST typing of PCR-positive throat swabs revealed a marked shift in genotype composition over time. In 2021, P1-2/ST14 and P1-1/ST3 were present at comparable frequencies (48.7% vs 44.5%), with a small contribution from ST30 (5.9%), whereas by 2023 and 2024 ST3 accounted for 65.8% and 80.1% of typed specimens and ST14 declined to 30.6% and 13.9%, respectively (**Table 4**). Untypeable P1–1 and P1–2 profiles remained uncommon in all three years, and genotype distributions are reported for specimens with successful P1/MLST typing.

**Table 4:** Genotype distribution of *Mycoplasma pneumoniae* PCR-positive clinical specimens, 2021–2024.

Year	Total (n)	P1–1 Subtypes			P1–2 Subtypes	
		ST3	ST30	Untypeable	ST14	Untypeable
2021	119	53 (44.5%)	7 (5.9%)	1 (0.8%)	58 (48.7%)	0 (0.0%)
2023	85	56 (65.8%)	0 (0.0%)	3 (3.5%)	26 (30.6%)	0 (0.0%)
2024	115	93 (80.1%)	0 (0.0%)	4 (3.5%)	16 (13.9%)	2 (1.7%)

Data are presented as n (%), where percentages are calculated relative to the number of PCR-positive specimens with successful direct P1/MLST typing (n) for each year. Untypeable indicates specimens for which a complete MLST type could not be assigned.

**Figure 3:**
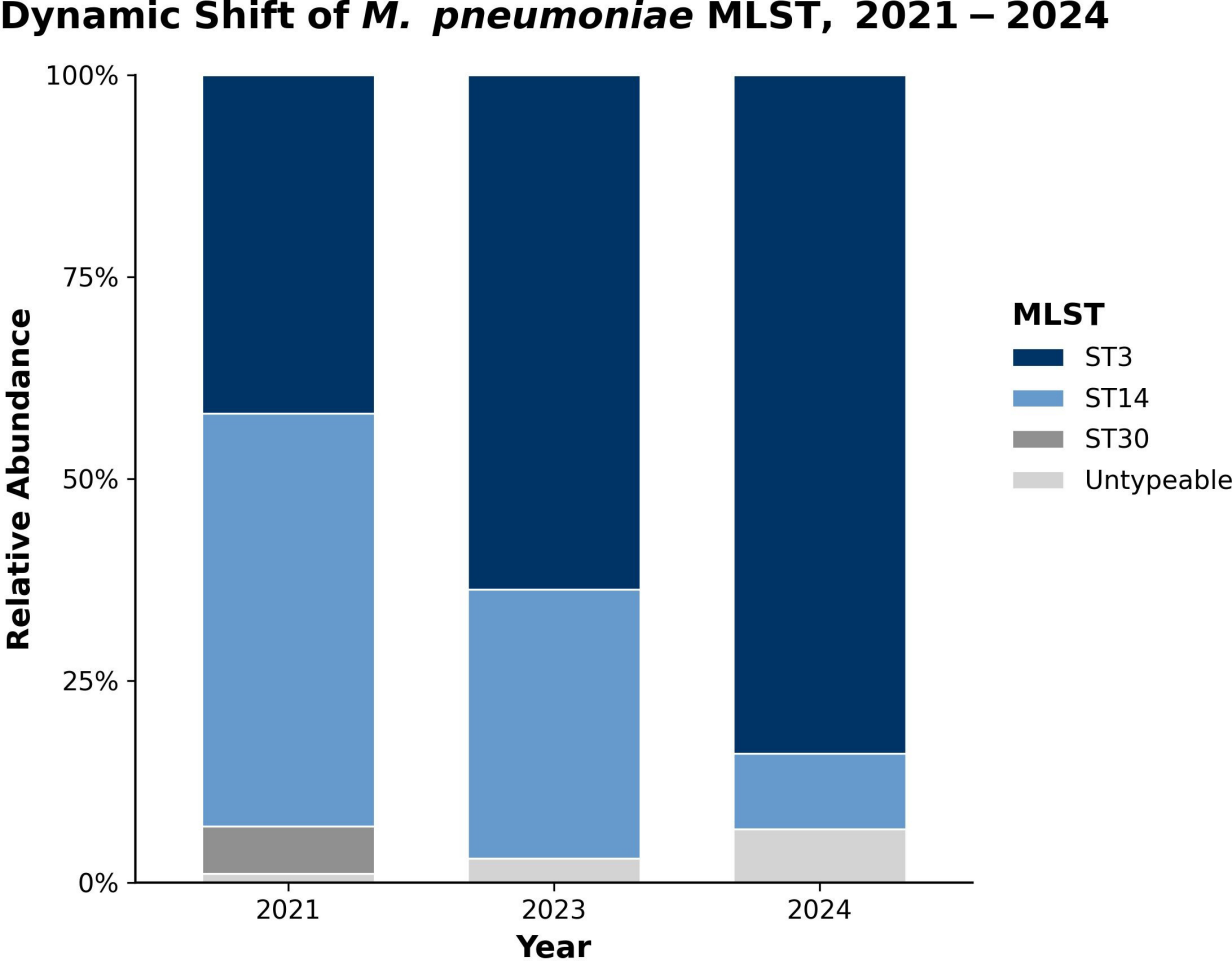
Distribution of *Mycoplasma pneumoniae* MLST genotypes, 2021–2024. The stacked 100% bar chart shows the relative abundance of major genotypes for isolates collected in 2021 (n=86), 2023 (n=66), and 2024 (n=75). A rapid increase in the proportion of the ST3 clone is evident over the study period, displacing the ST14 and ST30 clones.

To further characterize genotype dynamics during the resurgence, we performed P1 and MLST genotyping on all 227 cultured isolates. The isolate-based analysis showed a rapid shift in predominance (**Figure 3**, **Table 1**). In 2021, the *Mycoplasma pneumoniae* population was mixed, with P1-1 (predominantly ST3) and P1-2 (exclusively ST14) circulating at comparable frequencies (48.8% vs 51.2%). By 2023, P1-1/ST3 had become predominant (63.6%), reaching 84.0% (63/75) in 2024. Over the same period, P1-2/ST14 declined (33.3% in 2023 to 9.3% in 2024), and ST30 was not detected after 2021. A small number of untypeable isolates were observed (P1-1: 2 in 2023 and 3 in 2024; P1-2: 2 in 2024). Together with the specimen-level findings, these patterns support clonal expansion of the P1-1/ST3 lineage during the 2023–2024 epidemic in Jinan. Using the EC1/EC2-specific SNP panel, all 141 ST3 isolates (all P1-type 1) were classified as EC1, whereas all 73 ST14 isolates belonged to EC2. Thus, the post-COVID resurgence in Jinan reflects expansion of a P1-1/ST3 EC1/T1-3R lineage against a background of declining P1-2/ST14 EC2/T2–2 lineage.

### 3.4 Phylogenetic and genomic evidence for clonal replacement

To investigate the genomic epidemiology of the recent *M. pneumoniae* outbreak, we built a maximum-likelihood phylogeny from core-genome single-nucleotide polymorphisms (SNPs) for all 227 isolates. The core-genome tree resolved two major clusters that corresponded to the P1-type 1/T1-3R (EC1) and P1-type 2/T2-2 (EC2) lineages, in close agreement with P1 genotypes and EC1/EC2 clone assignments (**Figure 1**). Isolates from 2021 were scattered across both lineages, including a small subset of P1-type 1 isolates lacking the EC1/EC2 signatures that formed a T1-3-like branch, whereas almost all isolates from 2023–2024 grouped within the T1-3R (EC1) lineage and formed a compact, star-like cluster consistent with rapid recent expansion of a single clone. Notably, although both T1–3 and T1-3R subclades have been described in global datasets, all 141 ST3 genomes in this study carried the EC1 SNP signature and fell within T1-3R; we did not observe any ST3 isolates assigned to T1-3. The only non-EC1 type 1 genomes were six isolates sampled in 2021 (five ST30 and one MLST-untyped), forming a small T1-3-like branch that was not seen in subsequent years.

**Figure 4:**
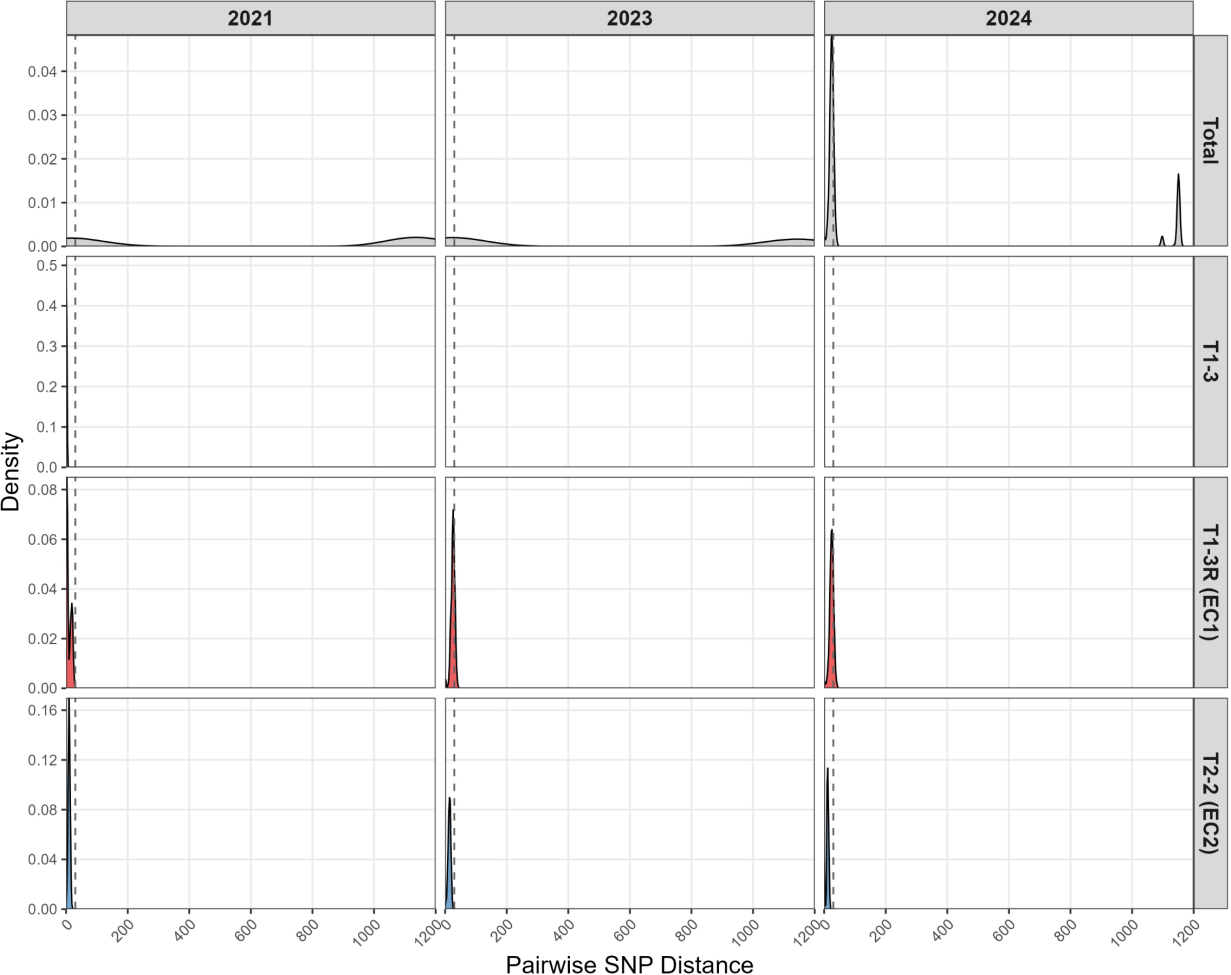
Pairwise SNP distance distribution by year and clade. Distributions of pairwise core-genome SNP distances stratified by collection year and phylogenetic clade. The 2021 panel shows a bimodal distribution, consistent with co-circulation of divergent type 1 (T1-3/T1-3R) and type 2 (T2-2) lineages. In 2023 and 2024, the distributions are dominated by a single sharp peak at low SNP distances, driven by the T1-3R (EC1) clade, indicating a recent clonal expansion, while T1–3 disappears and T2-2 (EC2) persists only as a smaller, more diverse subset.

Pairwise SNP-distance profiles quantitatively mirrored this pattern (**Figure 4**). In 2021, a bimodal distribution indicated co-circulation of two genetically distant lineages; by 2024, the distribution collapsed to a single narrow peak with most pairs differing by <30 SNPs, consistent with a highly clonal population. Taken together, these data indicate clonal replacement, with a relatively homogeneous T1-3R (EC1) lineage displacing previously co-circulating, more diverse lineages including T1-3/T1-3R and T2-2. In a broader context, a phylogeny incorporating 308 global strains placed the Jinan outbreak cluster closest to contemporaneous isolates from other Chinese cities (e.g., Beijing), supporting regional transmission rather than a novel introduction (**Figure 2**).

### 3.5 Transmission network analysis reveals a highly interconnected outbreak

**Figure 5:**
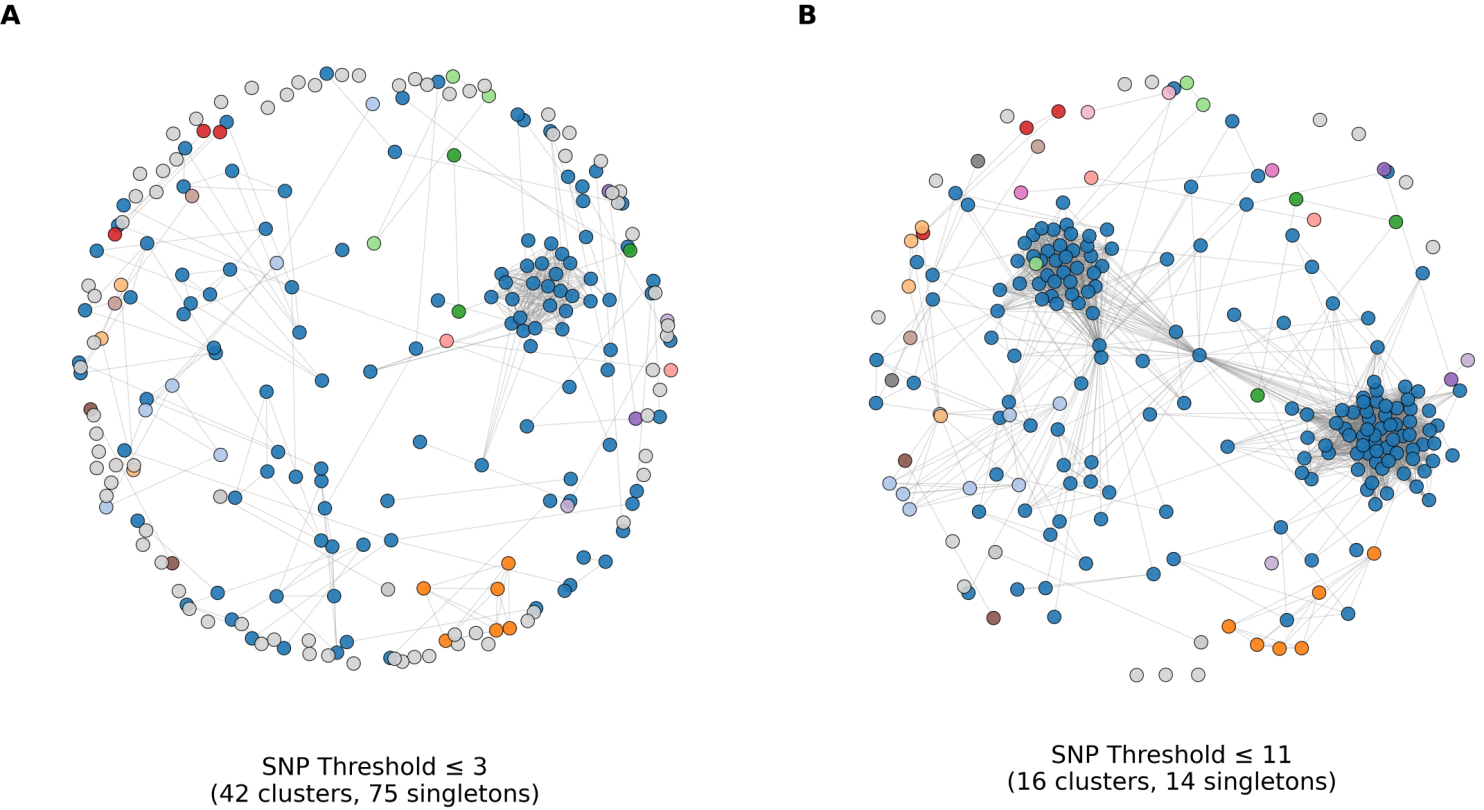
Genomic transmission networks at alternative SNP thresholds. Nodes are isolates; an edge links two isolates whose pairwise core-genome SNP distance is at or below the indicated threshold. Layout is force-directed. **(A)** ≤3 SNPs: fragmented network with 42 clusters and 75 singletons. **(B)** ≤11 SNPs: 16 clusters with a dominant supercluster and 14 singletons. Node colors match cluster membership defined at the ≤11 SNP threshold; gray nodes are singletons.

Pairwise SNP–based networks showed strong clustering (**Figure 5**). Using a strict threshold of ≤3 SNPs, the network broke into many small components (42 clusters) with numerous singletons (n=75), consistent with short, localized chains. At a more permissive but still conservative threshold of ≤11 SNPs, isolates merged into 16 clusters, including one dominant supercluster containing 168 isolates (74% of all samples). This pattern indicates that most isolates belonged to a highly homogeneous clone that was widely distributed in the population. In a slowly evolving, low-diversity pathogen such as *M. pneumoniae*, such large connected components at a ≤11-SNP threshold primarily reflect limited genomic diversity and recent clonal expansion, and do not imply direct epidemiologic linkage among all connected cases.

### 3.6 Pan-GWAS differentiates T1-3R/T2–2 while adhesion and CARDS toxin remain conserved

**Figure 6:**
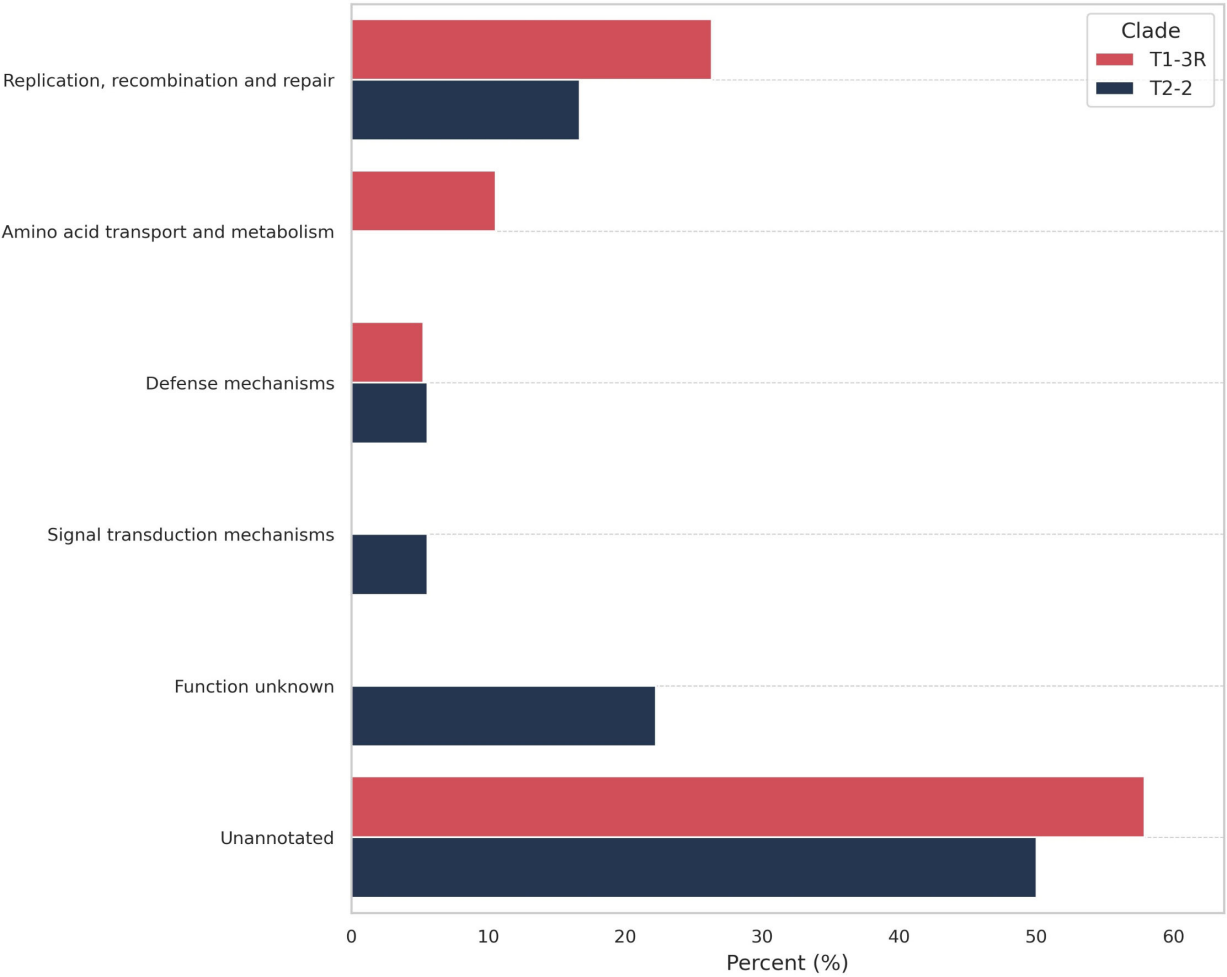
Functional comparison of differential genes in the T1-3R (EC1) (red) and T2-2 (EC2) (blue) clades. The chart shows the percentage of genes in key COG categories, highlighting a functional enrichment for metabolic and replication-related processes in the T1-3R (EC1) clade.

A presence/absence pan-GWAS identified 37 clade-associated gene clusters (FDR q<0.05), with 19 enriched in T1-3R (EC1) and 18 in T2-2 (EC2). Mapping these clusters to COG categories revealed clear functional skews (**Figure 6**): T1-3R showed a higher share of clusters annotated to “Replication, recombination and repair” and uniquely contained clusters in “Amino acid transport and metabolism.” Both clades contributed one cluster in “Defense mechanisms,” whereas a single “Signal transduction mechanisms” cluster was observed only in T2-2. Notably, a large fraction of significant clusters in both clades were “Unannotated” (i.e., without COG assignment), with a higher proportion in T1-3R (EC1). In contrast, “Function unknown” (COG assigned but with unknown function) was more represented in T2-2. Together, these patterns point to clade-specific differences in genome maintenance and metabolic modules while also highlighting the substantial fraction of genes that remain poorly characterized in *M. pneumoniae*.

Next, we surveyed virulence determinants across the 227 genomes using the Virulence Factor Database (VFDB), a curated resource widely applied for genome-scale virulence profiling. Across the 227 *Mycoplasma pneumoniae* genomes, HMW1 (MPN447), HMW2 (MPN310), HMW3 (MPN452), P1 (MPN141), P40/P90 (MPN142), P30 (MPN453), P65 (MPN309), and CARDS toxin (MPN372) were detected in all isolates.

### 3.7 Universal macrolide resistance contrasts with susceptibility to other antibiotics

All 227 isolates harbored the 23S rRNA A2063G substitution, the canonical marker of macrolide resistance. No other known macrolide-resistance substitutions in domain V (A2063C, A2063T, A2064C, A2067G, C2617A or C2617G) were observed. In line with this genotype, macrolide MICs were uniformly high—erythromycin >16 µg/mL and azithromycin 8–>16 µg/mL (**Table 5**)—well above the CLSI M43-A susceptible cutoff of 0.5 µg/mL. By contrast, tetracycline and levofloxacin MICs stayed within the M43-A susceptible range (≤2 and ≤1 µg/mL, respectively); no isolate exceeded these thresholds, indicating preserved *in vitro* activity in this cohort.

**Table 5:** MIC distributions and CLSI M43-A interpretive information for 227 *Mycoplasma pneumoniae* clinical isolates.

Antibiotic	Isolates ≥ M43-A R threshold^a^ (n/N, %)	Observed MIC Range^b^ (µg/mL)	M43-A interpretive info^c^
Erythromycin	227/227(100)	> 16	S ≤0.5; R ≥1
Azithromycin	227/227(100)	8 – > 16	S ≤0.5; R ≥1
Tetracycline	NA	≤ 0.125 – 0.5	S ≤2; R not defined
Levofloxacin	NA	≤ 0.125 – 1	S ≤1; R not defined

For macrolides, “R” follows CLSI M43-A proposed criteria (≥1 µg/mL). For tetracycline and levofloxacin, CLSI M43-A does not define resistance thresholds; therefore counts are not assigned (NA).

^b^Ranges reflect observed values across 227 isolates; “>16” indicates the highest concentration tested.

^c^CLSI M43-A provides susceptible cutoffs for all agents listed and proposed resistance cutoffs only for macrolides.

## 4 Discussion

Our investigation of the post-COVID-19 resurgence of *Mycoplasma pneumoniae* in Jinan reveals that this was not a simple epidemiological rebound but a clonal replacement. This selective sweep coincided with higher clinical severity indicators and occurred during a period when a P1-1/ST3 lineage displaced previously co-circulating variants, a pattern that was evident both among directly genotyped PCR-positive specimens and among cultured isolates. Because severity is influenced by many host and care factors and our study is observational, these parallel trends should be interpreted as associations rather than proof that the ST3 lineage itself caused more severe disease. High-resolution genomic analysis allowed us to relate pathogen fitness, host immunity, and antimicrobial resistance in the post-pandemic period.

The defining feature of the 2023–2024 epidemic was the predominance of the P1-1/ST3 clone, which rose from 44.5% to 80.1% among directly typed PCR-positive specimens and from 41.9% to 84.0% among cultured isolates between 2021 and 2024. This pattern is supported by our genomic data: the phylogenetic tree shows a compact, near star-like topology of recent isolates within the T1-3R (EC1) clade, and pairwise SNP distances contracted from a bimodal distribution in 2021 to a single narrow peak dominated by T1-3R (EC1) comparisons in 2023–2024. This is consistent with recent clonal expansion (Brown et al., 2016; Meyer Sauteur et al., 2025). To explore transmission at finer scale, we constructed SNP-threshold networks. At a ≤11-SNP cut-off, the network contained large connected components. While this threshold allows for comparison with recently reported genomic analyses of the Chinese epidemic (Jiao et al., 2025), we interpret these broad clusters with caution. Given the inherently slow substitution rate and low genomic diversity of *M. pneumoniae*, these large components probably reflect the general expansion of the dominant T1-3R lineage during the outbreak rather than definitive chains of direct person-to-person transmission. In contrast, a stricter ≤3-SNP threshold is more consistent with recent transmission within the community, although even pairs within this range may not always represent single-step transmission events.

While the “immunity debt” hypothesis explains the large pool of susceptible hosts available, it does not explain why this specific clone prevailed. The post-NPI environment likely increased selection for transmissible lineages, creating conditions favorable to spread, where a lineage with even a marginal advantage in transmissibility could increase in frequency and displace co-circulating variants. Our pan-genome analysis is compatible with a potential advantage, showing the dominant T1-3R (EC1) clade was enriched for genes involved in “replication, recombination and repair” (Meyer Sauteur et al., 2025). The observation of a similar P1–1 genotype dominance in a concurrent Beijing outbreak suggests a regional pattern rather than a purely local event (Chen et al., 2024; Jia et al., 2024). Comparable expansions of macrolide-resistant ST3 lineages have been reported across East Asia, and ST3 MRMP now forms a predominantly East Asian subclade encompassing China, Korea, Japan and Taiwan (Yun, 2025; Tewolde et al., 2025).

Our pan-GWAS indicated functional differences between circulating clades while key virulence determinants remained conserved. The enrichment of replication/recombination/repair genes in T1-3R (EC1) is consistent with evidence that diversification in *M. pneumoniae* concentrates around RepMP repeats flanking the P1 operon, where intragenomic homologous recombination generates subtype-linked polymorphisms (Kenri et al., 1999; Spuesens et al., 2009). In contrast, core terminal-organelle components—P1, P40/P90, P30, HMW1/2/3—were detected across our dataset, consistent with their roles in cytadherence, gliding and cell morphology (Romero-Arroyo et al., 1999; Vizarraga et al., 2020). The presence of CARDS toxin across all genomes further supports a shared virulence axis: CARDS toxin is an ADP-ribosylating/vacuolating toxin implicated in airway injury and inflammation (Kannan and Baseman, 2006). Taken together, T1-3R (EC1) may have greater DNA-maintenance capacity while retaining adhesion and toxin genes required for transmission. However, gene presence alone does not equate to virulence. Comparative “omics” work has shown subtype- and strain-specific differences in CARDS toxin expression despite highly similar genomes, and recombination-driven antigenic and phase variation in P1 and related adhesins can change surface exposure and host interaction, underscoring the need for transcriptomic and proteomic comparisons between clades (Lluch-Senar et al., 2015; Hakim et al., 2021).

Uniform macrolide resistance probably shaped the clinical impact of the epidemic, even if it did not determine which lineages became dominant. All 227 isolates harbored the well-established A2063G mutation and were phenotypically resistant to macrolides, consistent with surveillance across China showing resistance rates over 90% (Yan et al., 2024). At the genomic level, we focused on canonical domain V mutations in 23S rRNA to define macrolide resistance. Recent whole-genome studies, however, suggest that additional loci—including macrolide-specific efflux pumps and mutations in ribosomal protein L4 and other housekeeping genes—may modulate resistance in conjunction with 23S rRNA changes (Li et al., 2017; Wang et al., 2023). A systematic genome-wide dissection of such secondary mechanisms, ideally incorporating quantitative MIC data and longitudinal sampling, was beyond the scope of the present study. Since high-level resistance was already fixed in both the P1-1/ST3 and P1-2/ST14 lineages before 2023, it conferred no selective advantage for the clonal sweep. Instead, it may have contributed to poorer clinical outcomes. As the highly transmissible ST3 clone spread, uniform resistance likely reduced the effectiveness of first-line azithromycin and was associated with prolonged fever, extended hospital stays, and progression to severe disease in some patients, although our observational data cannot exclude confounding by other factors, necessitating a switch to second-line agents like tetracyclines. The complete susceptibility of all isolates to tetracycline and levofloxacin informs current clinical practice (Jia et al., 2024; Jiang et al., 2021).

This study illustrates the value of integrating genomic surveillance into public health practice. Traditional methods would have detected only an increase in cases; whole-genome sequencing revealed the underlying mechanism of clonal replacement. The genomic signature of the epidemic clone—P1-1/ST3, T1-3R (EC1) clade, and a characteristic virulence gene profile—serves as a genomic fingerprint for a high-risk lineage. This supports prospective genomic monitoring: tracking this fingerprint in new regions could serve as a prospective flag for emerging clusters (Meyer Sauteur et al., 2025), allowing timely clinical alerts and updated treatment guidelines. Such genomically informed strategies may improve outbreak detection and treatment alignment in the post-pandemic era.

This study has several limitations. First, as a single-center, retrospective study, it may be subject to referral bias, potentially over-representing more severe cases of MPP. Second, all genomic and genotype–phenotype analyses were limited to the 227 children with cultured isolates (28.5% of the 798 MPP cases), so lineages that grow poorly *in vitro* and patients without recoverable isolates may be under-represented, although P1/MLST distributions among isolates closely matched those obtained by direct typing of PCR-positive specimens (**Tables 4**, **1**). Third, our sampling periods were temporally asymmetric: isolates in 2021 were obtained over an 8-month interval, whereas 2023 and 2024 contributed only 2 and 3 months of sampling, respectively. Because *M. pneumoniae* epidemics can span multiple seasons within a 1–2-year wave (Wang et al., 2024; Wu et al., 2024), this uneven calendar coverage may inflate apparent genetic diversity in 2021 and reduce the precision of between-year comparisons of lineage frequencies and severity, even though all clinical and genomic summaries are stratified by year. Fourth, although we applied the same guideline-based definition of severe pneumonia throughout, we did not routinely capture all viral and bacterial co-infections, and we cannot fully exclude the influence of changes in admission or intensive-care practice over time. Prior pediatric studies have shown that co-infection with respiratory viruses or other bacteria can worsen the course of *M. pneumoniae* pneumonia, so part of the increase in severe cases may reflect unmeasured co-infections or shifts in clinical practice rather than lineage effects alone (Song et al., 2015; Li et al., 2022). Finally, the observed association between the P1-1/ST3 genotype and increased clinical severity is purely correlational and should not be taken as evidence that this lineage is intrinsically more virulent; establishing causality will require prospective studies and experimental validation in appropriate models.

## Data Availability

The data are now publicly available and searchable in NCBI. The sequencing dataset has been released (Released; release date: 2025-12-29) under BioProject accession PRJNA1337184 (“Genomic study of Mycoplasmoides pneumoniae”; 227 BioSamples and 227 SRAs).
